# Tumour Markers and Kidney Function: A Systematic Review

**DOI:** 10.1155/2014/647541

**Published:** 2014-02-06

**Authors:** Giuseppe Coppolino, Davide Bolignano, Laura Rivoli, Giuseppe Mazza, Piera Presta, Giorgio Fuiano

**Affiliations:** ^1^Nephrology and Dialysis Unit, University Hospital “Magna Graecia”, 88100 Catanzaro, Italy; ^2^CNR Institute of Clinical Physiology (IFC), 89100 Reggio Calabria, Italy

## Abstract

Tumour markers represent useful tools in diagnosis and clinical management of patients with cancer, because they are easy to use, minimally invasive, and easily measured in either blood or urine. Unfortunately, such an ideal marker, as yet, does not exist. Different pathological states may increase the level of a tumour marker in the absence of any neoplasia. Alternatively, low levels of tumour markers could be also found in the presence of neoplasias. We aimed at reviewing studies currently available in the literature examining the association between tumour markers and different renal impairment conditions. Each tumour marker was found to be differently influenced by these criteria; additionally we revealed in many cases a lack of available published data.

## 1. Introduction

Tumor markers represent useful tools in diagnosis and clinical management of patients with cancer, because they are simple to use, minimally invasive, and easily measured in blood and urine. More than 20 different substances (hormones, metabolites, enzymes, immunoglobulins, tumour associated antigens, and oncogenes) have been identified as tumor markers and are currently employed in clinical analysis. Some markers are positive in a single type of cancer, others are detectable in more than one type. The ideal tumour marker should be (a) positive in patients only if malignancy is present or in progress, (b) correlated with stage and response to treatment, (c) repeatedly detectable, and (d) easily measured [1]. Unfortunately, such an ideal marker, as yet, does not exist. Some markers play a role in the diagnosis of cancer but in particular are instrumental for the control of oncologic patients response to therapy. In this view a frequent approach to monitor these patients is the determination of changes in concentration of established parameters like tumour markers half-life (*t*_1/2_) and tumour marker doubling time [2]. Different pathological states may increase the level of a tumour marker in the absence of any neoplasia (false positive), while, in other cases, not every subject with cancer has abnormally high levels of the tumour marker usually associated with that neoplasia (false negative). Therefore, the most available tumour markers have insufficient sensitivity and specificity to be used alone for diagnosing or screening neoplasias but have to be combined with other diagnostic tools. In the last decade, international scientific societies of oncology have published guidelines for the appropriate use of tumour markers and guidelines to improve their correct use in prognostic studies, considering the evaluation of each single tumour marker [3, 4]. Nevertheless, the clinical use of tumour markers remains a controversial question for many reasons. Firstly, it should be emphasized that the majority of the markers are tumour-associated rather than tumour-specific. Additionally, many factors may lead to false positive and/or negative results. Biomarkers levels, in fact, may be modified by several tumor-independent physiological or pathological states, like menstrual cycle, pregnancy, metabolism, inflammation, and liver and/or renal function [5]. In this review we will examine literature on the association between tumour markers and different renal impairment conditions.

## 2. Kinetics of Serum Tumour Markers and Renal Function

Elevated levels of several tumour markers can be frequently detected in patients with impaired kidney function because their renal elimination is retarded or because a neoplasia is really present, given the higher risk of developing malignancies in these patients (e.g., because of altered immune-surveillance induced by the inflammatory state or by immunosuppressive therapies). Consequently, while cancer screening and surveillance are important in this population, the possibility of false positive results notably reduces the diagnostic value of those markers that are mainly eliminated by renal excretion [6–9]. Despite this significant complication, only few reports exist on tumour markers measurement and metabolism in patients with impaired renal function, including those with proteinuria, reduction of glomerular filtration rate (GFR) with chronic kidney disease (CKD), end stage renal disease (ESRD), on renal replacement therapy (RRT) by haemodialysis, peritoneal dialysis, or having had kidney transplantation. This information, in fact, is very important [10] because (a) GFR reduction can lead to impaired excretion of most tumour markers, (b) proteinuria may affect the metabolism and excretion of protein biomarkers, (c) the chronic inflammatory state associated with CKD can affect plasmatic and urinary levels of some markers, and (d) the markers with small molecular size could be eliminated by dialysis. As a consequence, levels of tumour markers can be higher in patients with renal dysfunction because of reduced urinary elimination or even lower because of removal by the dialysis procedure.

## 3. Methods Used for Literature and Selection Process

**Figure 1:**
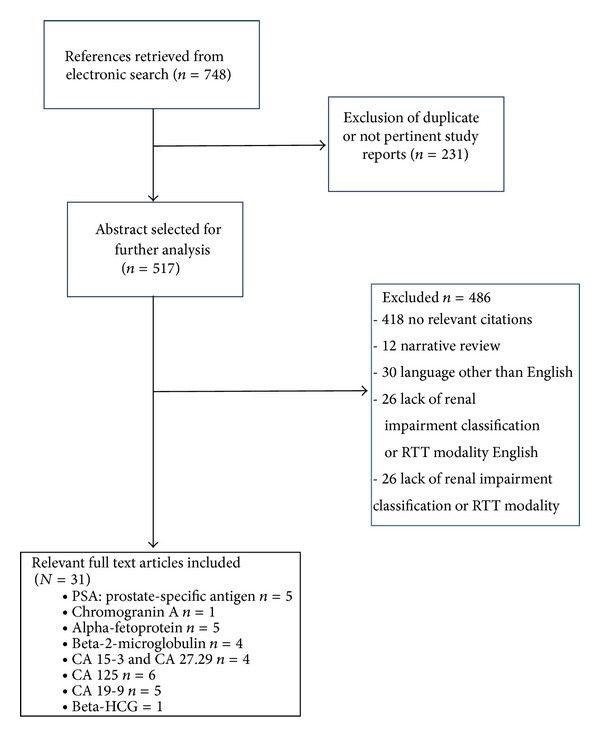
Flow diagram of the literature selection process.

We aimed at reviewing eventual studies currently available in literature, excluding those that examined the diagnostic and prognostic value of different types of tumour markers without keeping into account the severity of renal impairment, the presence and degree of proteinuria, and the type of RRT. Studies that examined the association between tumour markers and renal impairment stages were identified by a computerized research of all English-language articles in the electronic database PubMed. We conducted a systematic search of full text papers, published between 1990 and December 2012, by combining the following Medical Subject Heading (MeSH) terms: “tumour marker” or “neoplastic marker” or the name of single neoplastic marker combined with “renal disease” or “chronic kidney disease” or “renal failure” or “haemodialysis” or “peritoneal dialysis” or “renal transplant”. We considered all types of clinical studies, including parallel nonrandomized, randomized, and crossover trials, observational studies, and meta-analyses. Seven hundred forty-eight references were initially retrieved; two hundred thirty-one studies were excluded because they were duplicates or not pertinent with our topic. Four hundred eighteen references were discharged after full text analysis considered them to be not relevant, 12 being narrative reviews without new data to be considered, 30 being in languages different from English, and 26 having no data available on renal impairment or RTT modality. Thirty-one full text articles were therefore included in the final analysis.  Figure 1 depicts a flow chart of the selection process.  Table 1 resumes the main variations of tumor markers levels under different renal impairment conditions.

### 3.1. PSA: Prostate-Specific Antigen

PSA is a 33 kDa androgen-regulated serine protease (glycoprotein) secreted by prostate epithelial cells that takes part in the liquefaction of seminal fluid [11]. It is released in the blood in two predominant forms: free, noncomplexed PSA (fPSA) with a molecular mass of *∼*33 kDa, and complexed PSA (cPSA), a stable complex with alpha-1-antichymotrypsin (*∼*90 kDa). The routinely immune-detected total PSA (tPSA) is the sum of fPSA and cPSA [12]. The PSA discovery and its availability as a routine laboratory test have revolutionized the diagnosis of prostate cancer, the most common malignancy and the second leading cause of cancer-specific death in the male. Serum levels of PSA are commonly used for diagnosis, screening, risk stratification, staging, monitoring of treatment outcomes, and recurrence detection [13]. However, PSA levels are considerably altered in course of CKD. In 2009 Bruun et al. evaluated the levels of PSA; fPSA; and cPSA of 101 men (median age of 57) with varying degrees of renal failure without any history of prostate cancer or urinary tract symptoms and compared them with data of 5264 healthy control men from the European Randomized Screening for Prostate Cancer (ERSPC) study. GFR was measured with iohexol clearance and the result was that men with CKD or impaired renal function have significantly higher levels of fPSA compared to controls (*P* < 0.001) [14]. These results were confirmed by the National Health and Nutrition Examination Survey (NHANES) in a larger study published in 2010 on 3782 males aged ≥40 years, enrolled between 2001 and 2006 [15]. In this study all possible confounding factors were excluded: like current prostatitis, rectal examination in the previous week, prostate biopsy in the previous 4 months, cystoscopy, and history of prostate cancer and a multivariate linear regression model was performed to determine the adjusted relationship between GFR and PSA outcomes (tPSA, %fPSA) after adjustments for age and race. GFR resulted negatively correlated with fPSA thus suggesting caution in interpreting fPSA levels in patients with renal impairment. Indeed, in patients with GFR <90, the accuracy of fPSA in discriminating between prostate cancer (Pc) and benign prostatic hypertrophy (BPH) is limited [14]. Both hemodialysis and peritoneal dialysis are believed to influence the PSA-based results but few studies have been as yet published. Arican et al. and Arik et al. both published two papers about changes in tPSA in patients undergoing dialysis treatment; the first enrolled 50 patients on HD, of which only 26 men in standard HD modality, and analyzed their levels of CEA, CA-125, CA15-3, b-HCG, PSA, and AFP after the dialysis session; the second enrolled 35 HD patients and 35 healthy volunteers and assayed CA 19.9; CA 125; CEA, AFP; and PSA. In these studies PSA levels were not altered [16, 17]. Tzanakis et al. carried out a multicenter trial enrolling 63 haemodialysis patients (aged 33–86 years old) and 729 healthy control subjects. In dialysis patients, tPSA was measured before and after a dialysis procedure performed with standard low flux membranes. The levels of tPSA were lower in dialysis patients than in the general population [18]. The dialysis procedure itself does not seem to remove a significant amount of this marker and postdialysis PSA levels increase proportionally to haematocrit. In 2003 Bruun et al. enrolled 20 HD men (median age 66) and 25 men in continuous ambulatory peritoneal dialysis (CAPD) comparing their tPSA; cPSA; and fPSA levels with data of 3129 healthy men (median age 57) from 3 different studies [19]. They also excluded from all groups men with diagnosis of prostate cancer and age <40 years. Indeed in this paper, patients were all well described for HD and CAPD technical features: for HD analysis a low-flux membrane was used and it was performed in standard 4-hour sessions 3 times a week; authors specified also Kt/v, dialysate flow, and dialysate total amount; CAPD daily treatment regimen was performed in four to five exchanges of 2–2.5 L of dialysis fluids, resulting in 8–12.5 L of dialysate per 24 h. Tests were performed before and after dialysis session. Finally authors did not specify ESRD causes. For HD and CAPD patients were measured for residual renal function with plasma clearance of iohexol. fPSA was *∼*40% higher both in HD and in CAPD patients (*P* < 0.01) while tPSA did not differ significantly. In the same year Sumura et al. published a study that included 41 HD men (excluding men with f prostate cancer, prostatic surgery, current urinary tract infection, and urinary retention) and measured predialysis tPSA levels. The particularity of this study is that patients were treated with high flux membrane and investigated also with DRE, TRUS and in the case of positivity to first level diagnostic tests were performed also CT and MRI. PSA resulted in an appropriate marker also in HD population, but this remains a small sample study without a control arm [20]. In 2006, a large Japanese study compared 1250 men on HD aged >50 years with 1007 males with normal renal function aged >55 years and found that serum total PSA levels were slightly higher in HD group, as well as correlating with the incidence of prostate cancer [21]. Data were collected both before and after haemodialysis sessions but Wada et al. did not specify membrane, time, and modality of dialysis treatment [22]. The only systematic study was made by Tarhan et al. in 2007 and included 36 patients (exclusion criteria were history of prostatic cancer, surgery and biopsy, DRE, acute urinary tract infections retention and catheterization, use of alpha-reductase inhibitors, and history of liver disease) treated with standard HD modality (thrice weekly for 3- to 5-hour duration with a blood and dialysate flow rates from 200 to 400 mL/min and from 500 to 600 mL/min, resp.). The membrane was a low-flux polysulfone HD membrane. The study was conducted to detect tPSA, fPSA, and cPSA values in plasma and ultrafiltrate, in addiction authors calculated the difference between pre- and postdialysis levels (Δ concentration) and estimated the hematocrit's influence. This study highlighted that, after HD session, cPSA and fPSA significantly increased (*P* < 0.05) while dialysis implicates a non-significantly tPSA increase, perhaps due only to hemoconcentration [23]. Summarizing our analysis of the literature about PSA, it emerges that it is a useful diagnostic tool in nondialysis CKD, HD, and in patients who underwent CAPD, but in these groups caution is advised because plasmatic levels can be influenced by GFR and the residual renal function. Particularly in HD, plasma concentrations of tPSA, fPSA, and cPSA vary whether measured before or after the dialysis session and based on the type of membrane used or on dialysis modality. Therefore, more detailed studies should be conducted to better determine the weight of these variables. Finally PSA levels have not yet been evaluated on large population before and after kidney transplantation. Bruun et al. studied only 14 patients with immediate onset of renal function after renal transplantation. Blood samples were obtained before and at regular intervals up to 160 hours after transplanted kidney reperfusion. fPSA and tPSA returned to levels comparable to patients with normal renal function. Results verified the hypothesis that PSA forms are eliminated from the blood circulation by glomerular filtration and severe renal failure influences the levels of proteins in serum but the number of subjects enrolled is not sufficient to verify specific alterations of PSA levels in transplanted patients [24].

### 3.2. Chromogranin A

Chromogranin A (CgA) is a 49 kDa acidic hydrophilic protein synthesized in the chromaffin granules of the neuroendocrine cells and is traceable in the blood of healthy subjects at concentrations of less than 30 ng/mL [25]. Elevated levels of CgA in serum are detectable in patients affected by neuroendocrine (NE) or carcinoid tumors, pheochromocytoma, neuroblastoma, small cell lung cancer (SCLC), and prostate cancer [26]. Recently CgA resulted also as a useful biomarker in hepatocellular carcinoma (HCC) screening, representing a complementary test when the levels of *α*-FP are not sufficiently diagnostic (<200 ng/mL) [27]. Serum CgA determination can also be used to predict the progression or regression of NE tumors during the treatment [28]. However, CgA elevation has been rarely reported also in patients without cancerous conditions, such as essential hypertension, so that the possibility of false positive results should be considered [18, 25]. Many recent studies pointed the possible role of CgA as a “stress hormone” involved in hypertension and renal damage physiopathology [29]. In patients with reduced renal function a rise in serum CgA levels can be observed probably as the consequence of reduced renal degradation [30]. Hsiao et al. first analyzed CgA levels in a prospective study including 37 healthy controls and 105 patients with different renal impairments: 5 transplant recipients, 8 affected by glomerular disease (serum creatinine between 1.2 and 2 mg/dL), 30 mild to severe renal disease (serum creatinine between 2 and 7.5 mg/dL), and 62 ESRD subjects (serum creatinine < 7.5 mg/dL) were also stratified according to the causes of renal failure and divided according to creatinine levels. Indeed, to define the influence of HD or CAPD authors compared 5 subjects already on a thrice weekly HD regimen against 4 subjects on CAPD. This trial showed that CgA is strictly correlated with the degree of renal impairment, independently of CKD etiology, blood pressure, hyperparathyroidism, and sympathetic-adrenal activity [31]. This biomarker's levels were higher in dialysis patients compared to predialysis ESRD subjects, but there were not any statistically significant difference between CAPD and HD. In 2001 Tramonti et al. carried out a trial including 102 CKD patients excluding subjects with signs and/or symptoms of neuroendocrine cancers. They measured GFR by the bladder cumulative method, using 99mTc-DTPA as a tracer. This study first demonstrated the relation between serum levels of CGA and GFR reduction, in particular when it drops below to 40 mL/min there occurs a progressive increase in serum values of CGA (*P* < 0.001) thus in CKD patients CgA levels may also be up to 22-fold more [32]. Finally, in 2010 Castoldi et al. performed a study with the aim to evaluate oxidative stress and CgA levels in uraemia and dialysis. They measured biomarkers of oxidative stress like –SH, 8-OHdG, and ox-LDL and CgA in 89 subjects of which 21 CKD, 17 CAPS, and 51 HD and in 18 with normal renal function. All patients with impaired renal function had a CgA increased level (*P* < 0.01) thus confirming the relation between GFR degree and CgA rising [33]. Moreover authors suggested that this event might also be explained by an increased production of CgA as an oxidative stress factor itself, in particular it could be a uremic toxin [33]. Concluding respect to CgA we can assert that it is not a good tumour marker in patients with CKD especially when GFR is lower than 40 mL/min. In dialysis its levels are usually increased and there are no differences with respect to dialysis mode.

### 3.3. Alpha-Fetoprotein (AFP)

Alpha-fetoprotein is a 65 kDa embryo-specific protein normally produced by the fetal liver and yolk sac (1-2 months) and subsequently mainly by the adult liver, whose levels gradually decrease after birth [34]. In healthy adults its function is not known [35]. Normal values range between 10 and 20 mcg/L. In adults, the levels of AFP increase over 500 ng/mL in (1) hepatocellular carcinoma (HCC), (2) germ cell tumours (it is elevated also in 80 to 85 percent of men with nonseminomatous germ cell tumors), and (3) metastatic cancer in the liver originating from other primary tumours elsewhere [36]. AFP levels are not modified by the presence of chronic renal insufficiency, ESRD, or RRT, including haemodialysis, peritoneal dialysis, and renal transplantation [37].

### 3.4. Beta-2-Microglobulin (b2m)

Wild-type beta-2-microglobulin (b2m) is the 11.8 kDa noncovalently bound light chain of the major histocompatibility complex class I (MHC I). Physiologically, it has an essential role in chaperoning assembly of the complex for antigen presentation thus is ubiquitously present on the surface of all nucleated cells. Indeed, b2m is secreted in most body's biological fluids like urine, blood, and synovial fluid [38]. The normal serum b2m concentration range is 1.5–3 mg/L, typically every day 2.4 mg/kg is produced [39]. Higher serum levels suggest an increased production that can occur in (1) lymphoproliferative diseases, such as multiple myeloma, beta-cell chronic lymphocytic leukemia, Hodgkin's disease, and non-Hodgkin's lymphoma, (2) inflammatory conditions, such as systemic lupus erythematous, rheumatoid arthritis, Sjogren's syndrome, and Crohn's disease, (3) some viral infections, such as cytomegalovirus, non-A and non-B hepatitis, and mononucleosis [40]. Concerning its use in oncology b2m levels correlate with the disease stage and poorer prognosis in patients with multiple myeloma or chronic lymphocytic leukaemia and it is the most important predictor of treatment-free survival and overall survival of patients affected by lymphocytic leukaemia and generally in most of lymphatic neoplasia [41, 42]. Recently there are new emerging hypotheses for the role of b2m in breast, gastric, and other tissues metastasis and lethality, therefore b2m may be subject of future target therapy in cancer research [43]. The pathological condition which definitely determines the most significant increase in the levels of b2m is renal failure, so much so that this is now considered one of the main so-called “uremic toxins” belonging to the category of “middle molecule”. B2m is certainly one of the most frequently studied compounds in ESRD and dialysis patients and about this subject there are many studies in the literature. In kidney disease and dialysis b2m levels are found correlated with cardiovascular risk and all-cause mortality [44, 45]. Usually, in course of impaired renal function the range of b2m is 20–50 mg/L and it is possible in exceptional case to note levels higher than 100 mg/L. Levels of b2m begin to rise dramatically as the GFR drops below 40 mL/min [46]. Its deposition in joints, synovia, cartilage, and bones and subsequent formation of b2m-positive amyloidal fibrils are responsible for the pathogenesis of dialysis-related amyloidosis (DRA) characterized by local inflammation with secondary destruction [47, 48]. The fundamental reason to explain this phenomenon is the reduction of renal catabolic process: normally, as a result of its low molecular weight, 95% of all free b2m is rapidly eliminated by glomerular filtration; proximal tubular cells uptake 99.9 percent of this filtered amount by megalin-dependent endocytosis; moreover tubules contribute to its destruction, thus the GFR reduction and tubular activity reduction make that b2m serum concentration markedly increase in patients with CKD [49]. Further confirmation to which degree of successful transplant contributes to the reduction of signs and symptoms of DRA in relation to the decrease in plasma levels of this substance [50]. Nevertheless, retention is not the exclusive factor involved in CKD high b2m concentration [41]. Furthermore an enhanced production can be triggered in response to many other factors like systemic inflammation, acidosis, calcitriol or other drugs treatment, and above all dialysis procedure. Many studies *in vivo* and *in vitro* try to explain the b2m production; for example, it is thought that it is released by circulating monocytes. In particular in HD they would be stimulated by contact with nonbiocompatible membranes such as cuprophan. This is at present a very hot topic and of course requires more extensive research [51]. Highly permeable HD membranes are able to remove significant amounts of b2m whereas conventional low-flux membranes are impermeable for the molecule thus in the case of high-flux HD b2m levels are much lower [52]. The usefulness of b2m as tumour marker is limited in the presence of CKD, since the impairment of renal function may increase b2m levels in an unpredictable way. In 2009 Delgado et al. performed an interesting trial with the aim to evaluate the GFR burden on b2m prognostic value in a cohort of 450 subjects affected by chronic lymphocytic leukaemia; they used a multivariate analysis method displaying a significantly better prognostic value of this marker adjusting the value for the GFR [53]. In this case when using b2m as a tumour marker it is appropriate to adjust its values for the degree of deterioration of renal function calculated with GFR.

### 3.5. CA 15-3 and CA 27.29

CA 15-3 and CA 27.29 detect circulating MUC-1 (mucin-1) antigen in peripheral blood [54]. MUC1 has a core protein mass of 120–225 kDa, which increases, to 250–500 kDa with glycosylation. CA 15-3 and CA 27.29 are, substantially, the same molecule but measures of different epitopes of the same protein antigen product of the MUC1 gene. Normal serum levels of CA-15-3 and CA-27.29 are less than 31 and 38 units/mL, respectively. MUC-1, which is expressed on the apical borders of secretory epithelial cells, has a protective function because of its ability to bind pathogens [55]. Overexpression, aberrant intracellular localization, and changes in glycosylation of this protein can be found in most human carcinomas [56]. While several studies support the prognostic relevance of MUC-1 in early stages of the breast cancer, there are no data suggesting that MUC-1-based serum tumour markers are helpful in guiding treatment decisions [57]. Serum levels of CA 15-3 and CA27.29 are not useful for breast cancer diagnosis, but their use is recommended in metastatic breast cancer treatment, since they reflect the course of disease in 75 to 90 percent of patients undergoing therapy [58]. However, the routine use of serial tumour measurement of these markers in the posttreatment surveillance of women with breast cancer is controversial [59–61], particularly in patients with CKD and RRT. Filella et al. did not observe any change in these conditions [62], while Zeferos found that CA-15.3 levels were significantly higher in patients on haemodialysis as compared to healthy volunteers and successfully transplanted patients [63]. Tzitzikos et al. confirmed that haemodialysis patients have higher level of CA 15-3 [64]. Xiaofang et al. did not report differences in CA 15-3 serum levels between uremic and control patients [65]. Giving the controversial results obtained in these studies, CA 15-3 cannot be considered sufficiently reliable in renal patients for diagnostic purposes.

### 3.6. CA 125

CA 125 (cancer antigen 125 or carbohydrate antigen 125) is a 90 kDa membrane protein with a single transmembrane domain, which is a member of the mucin family glycoproteins. It is a component of the ocular surface, respiratory tract, and female reproductive tract epithelia [66]. Normal values range from 0 to 35 *μ*g/mL [67]. It is increased in a variety of benign conditions like endometriosis, uterine leiomyoma, cirrhosis, pelvic inflammatory disease, pleural or peritoneal diseases, and malign conditions like ovarian, endometrial, breast, lung, and pancreatic cancer [68–70]. During the last 20 years, CA 125 has been used as a well-established marker for diagnosis of ovarian cancer [71]. In fact, serum CA 125 values are elevated in over 80 percent of women with ovarian cancer [72]. The average reported sensitivity for early stage disease is 50 percent in stage I and 90 percent in stage II [73], but the specificity is limited, because CA 125 levels are elevated in approximately 1 percent of healthy women [74], and fluctuate during the menstrual cycle. Mean CA 125 levels also vary with ethnicity and smoking status and increase with aging [75]. The metabolism and clearance of CA 125 are not well understood. Menzin et al. demonstrated that even advanced renal insufficiency is not always associated with significant elevation of CA 125 [76]. The development of renal insufficiency during treatment for ovarian cancer does not alter the serum levels of CA 125, as well as the haemodialysis procedure [77]. In peritoneal dialysis, CA 125 is elevated within 2 months after peritonitis and after PD catheter placement [78]. In these cases serum CA 125 level could be considered, with some caution, as a nonspecific marker of peritoneal inflammation [77]. CA 125 serum levels are not influenced by kidney transplantation [77].

### 3.7. CA 19.9

Carbohydrate antigen 19.9 or CA 19.9 is a 72.2 kDa tumour-associated carbohydrate antigen, initially found in patients with colon cancer and pancreatic cancer. It is mainly responsible for adhesion of human colon, pancreas, and gastric cancer cells to the endothelium [79]. Increased levels of CA 19.9 are also found in nonmalignant conditions, such as diseases of the bile ducts and liver [80]. Guidelines from the American Society of Clinical Oncology discourage the use of CA 19.9 as a screening test for cancer, particularly in pancreatic cancer as the marker holds a sensitivity and specificity of 80 to 90 percent, respectively [81]. However, the diagnostic performance of the biomarker is closely related to tumor size [82]. Serial monitoring of CA 19.9 levels is useful to follow up patients after surgery and those who are receiving chemotherapy for advanced disease [81]. In the literature, there are discordant data regarding CA 19.9 during renal disease. Filella et al. and Zeferos et al. did not notice differences in patients with chronic renal failure compared to normal subjects [62, 63] while Xiaofang et al. [65] and Arik et al. found increased levels of CA 19.9 in chronic kidney disease patients. Therefore, the use of this marker is not recommended in patients with impaired renal function [17].

### 3.8. Human Chorionic Gonadotropin (hCG)

Human chorionic gonadotropin (hCG) is a glycoprotein of 36.7 KDa produced in pregnancy [34]. Normal values in man and nonpregnant woman is <5 mIU/mL. hCG is an important tumour marker for gestational trophoblastic disease (GTD) and testicular cancer and is particularly helpful for GTD diagnosis, staging, followup, and recurrence rescue [83]. The production of hCG in testicular cancer depends in part upon the histological type. hCG is produced by nonseminomatous germ cell tumours that are comprised of pure or mixed embryonic carcinoma or choriocarcinoma and by 15 to 25 percent of seminomas that have either nonseminomatous elements or, more commonly, syncytiotrophoblast cells [33]. Among seminomas, the likelihood of an elevated serum hCG varies with disease severity, from 10 to 20 percent in earliest stages and from 30 to 50 percent in disseminated disease [84]. An increase in serum hCG correlates with the tumor mass [85]. Results from these studies indicate that kidney uptake is quantitatively relevant and both renal parenchymal metabolism and urinary excretion contribute to the elimination of hCG [86]. Consequently, the interpretation of hCG levels in CKD patients and dialysis patients is difficult, because its levels are normally elevated in these conditions [87].

## 4. Future Perspectives

Tumor markers, commonly used to assist in making a diagnosis and determining a prognosis, may result, in certain conditions, as false negatives or false positives. In our literature review we focused on the influence of different levels of altered renal function or on cases of renal replacement therapy (haemodialysis or peritoneal dialysis) or kidney transplant. Each tumour marker may be differently influenced by these conditions; importantly we revealed a lack of conclusive published data for some of these markers.

## Figures and Tables

**Table 1:** Summary of main variations of tumor markers levels in CKD, dialysis, and kidney transplantation.

	CKD	Hemodialysis	Peritoneal dialysis	Kidney transplantation
Alpha-fetoprotein (AFP)	=	=	=	=
Beta-2-microglobulin (b2m)	↑	↑	↑	↑
Beta-HCG	↑	↑	—	—
CA 15-3 and CA 27.29	↑	↑*; =*	—	=
CA 125	=	=	↑ In case of peritonitis or PD catheter placement	=
CA 19-9	=*; ↑*	—	—	—
Total tPSA	=	↓	=	—
Free fPSA	↑	↑	—	—
Chromogranin A	↑	↑	—	↑

= : unvaried with respect to patients with normal renal function; ↑: increased; ↓: decreased; —: no sufficient data; *see text.
